# In the Wards at the Royal Free

**Published:** 1889-10-19

**Authors:** 


### THE HOSPITAL WORKSHOP.

### No. XI.—IN THE WARDS AT THE ROYAL FREE.

(BY OUR SPECIAL COMMISSIONER.)

This hospital, facing the Gray's Inn Road, was built in 1842, on the site of an old barracks, of which a portion is still retained in front of the building. The committee, however, is making an effort to reconstruct the whole of that side, whereby room will be afforded for a children's ward, for surgery, dispensary, and other needful additions. At present a striking old gateway leads into a square, which forms a pleasant airing-ground for patients, ornamented as it is with a grass plot, and shaded by rustling Lombardy poplars. Under the guidance of one of the resident medical officers your commissioner was enabled to make a tour of this most interesting hospital.

The wards, which are lofty and well ventilated, are of convenient size, holding, on an average, about sixteen beds apiece. Their general look is comfortable and homely, an effect which is partly due to the cheerful red striped counterpanes. There are many pictures on the walls, and the ground floor, in particular, possesses some handsome oil paintings given by well-known artists. Plants and flowers are abundant; a few birds enliven the scene, while the "hospital dog," a pug belonging to one of the nurses, exercises a general supervision over the building. Each ward is provided with an invalid couch, the gift of various friends.

Admission is entirely free, as intimated by the name of the institution; and the patients, with but few exceptions, belong to the poorest classes. A great number of accidents are brought hither for treatment. Railway casualties are specially common, since this is the nearest large hospital to the main stations of the Midland at St. Pancras, and of the Great Northern and the Metropolitan at King's Cross.

In one of the surgical wards is a cabman who was admitted a few days ago with concussion of the brain, caused by a fall from his cab. He had come back to the yard from his day's work, and was folding up some things on the seat of the "hansom," with his back to the shafts, when a stableman moved the cab unexpectedly, and he was thrown to the ground. Next day, on admission, he had recovered consciousness; his pupils were unequal, a condition which still remains, and he was suffering a good deal from shock. He tells us, in answer to enquiries, that he has followed the same employment for twenty-seven years. The only other accident he has had was a fall from a loft ten or twelve years back, when his thumb was laid open and the drum of his left ear injured, which has rendered him rather deaf on that side. He adds that he now feels much the same sort of sensation in the right ear. He has not saved money, and seems to think that quite excusable because he has brought up a large family, neither does he belong to a benefit society. Has always enjoyed good health, a somewhat remarkable fact in a man who has driven a cab for more than a quarter of a century. Cabmen have a hard life, and suffer much from rheumatism and lung affections, brought on by constant exposure to all weathers. It is to be feared, moreover, that their habits, as a class, are not of the steadiest. One can easily understand why this should be so. Take, for instance, the case of a man cramped for many hours on the box of a night cab in the depth of winter. It is no wonder he should seek the readiest stimulant to his circulation at hand; nor is he likely to know that alcohol, after the first flush, actually lowers the temperature of the body.

The "Rhodes" is a square-shaped and cheery-looking ward. Here is a sailor sitting up in bed busily at work upon some kind of fringe of his own designing. His case is a curious one. The surface of the body generally is blue and mottled, and the extremities are cold. All feeling is lost down the front of the shins and along the insteps. One noteworthy fact is his statement that although his feet are cold by day they are warm by night. A native of Aberdeen, he has been at sea for twenty years, chiefly in sailing ships, and much of the time has been spent in hot climates. His disease resembles, in some of its symptoms, the "beri-beri" of the tropics.

In the male accident ward is a man of thirty-two, with his left hand fastened on a splint. The thumb and first two fingers of that side have been cut clean off by a two-foot circular saw. There was not much pain at the time; but he says it felt like a sudden blow, and the hand became numb. He has been working at the trade for fourteen years and "never had a scratch before." At the time of the occurrence he and his mates were cutting up short lengths of larch. When the bark is taken off that wood the log becomes very slippery, and it is then dangerous timber for the sawyers to handle. Properly speaking, the length to be sawn should be first squared with an axe; but as there was a press of work to get through, that precaution had been neglected. So that, while pushing a "stick" into position, his hand slipped right on to the saw, and the mischief was done in an instant. This man belongs to the Hearts of Oak Benefit Society, and he is entitled to eight shillings a week sick pay. He says that most sawyers belong to a friendly society, and it is worthy of remark, in passing, how providences of this kind are the rule in some trades while they are the exception in others. This patient is confident he will be able to work well enough with what is left of his hand, and is thankful the injury has not happened to his right. He will get no compensation from his employers, who "never give anything unless the matter is taken into court," but they undertake to provide work as long as they have any to give. Such a one-sided arrangement can hardly fail at times to be very unjust. It is pleasant to hear that this poor man will receive what he calls a "good gathering" from his fellow-workmen, who always get up a subscription in cases of this kind. The hand is dressed in lint and wool, prepared with corrosive sublimate and known as "Salalembroth" These materials, which are stained blue for the sake of distinction, are widely used in hospital practice.

In the next bed is a fine muscular young man, a railway excavator or "navvy" by occupation. While working at a tunnel a large mass of clay, four or five tons in weight, fell down suddenly, knocked him backwards, and completely buried the lower part of his body. It was nearly half an hour before the fallen earth could be broken up sufficiently to get him away. The accident happened at Oakley Park, on the Great Northern line, and he was brought to the hospital in a cab, thus entailing a painful journey of several hours' duration. On admission it was found that both bones of the left leg were broken, and there was a dislocation of the right foot above the ankle. He is doing well and most likely will recover with perfect use of his lower limbs.

A convalescent is sitting on an easy-chair in the ward, waiting to report himself to his surgeon. He is a groom, and tells his tale with voice and manner rather as if he was rehearsing evidence to give in court. "On the 16th October last I was sitting down behind a horse having my breakfast in the stable, about nine in the morning. The next horse flew at the one in front of me, and, although he was a very quiet horse, he couldn't stand that, but kicked out and struck me on the head, and then I didn't know any more for ten days." At the hospital he was found to have a compound fracture over the left eyebrow, going right through the skull and exposing the brain. The loose fragments of bone were taken away and a dressing applied. The wound is now healed, but shows a scar in which the little finger can be laid, and the vessels of the brain can be felt beating underneath. He was in bed for two months, and ever since the accident has been subject to fits. These come on usually at night, and just before he gets unconscious he feels a numbness in the hands and feet. He bites his tongue and foams at the mouth. This may be a case for modern surgery, where the removal of a cicatrix or "scar" from the brain substance will cure the epilepsy, which is here clearly a consequence of the injury. When asked if he got any compensation, he replied, "Master gave £2 my missus got off of him. I'm in a club, sir, the Foresters. They paid me 12s. a week for six months, and now I get 6s." Very few of his calling belong to any society, although their wages are good, a horse-keeper, for instance, receiving about 35s. a week.

The operating theatre is conveniently placed in a central position on the ground floor. It is well lighted by side windows and top skylight. At the time of our visit one of the surgeons was operating upon a lad suffering from diseased bones of the foot. After scooping out every bit of affected tissue, the cavity was stuffed with lint soaked in pure carbolic acid, a plan that is said to give excellent results. There were present some half-dozen lady students, of whom about thirty are attached to the hospital, and who fill the posts of dressers and clinical clerks. The operating table is worthy of mention. It is the invention of a former surgeon, and its details are worked out in a wonderfully ingenious manner. By a simple arrangement the ordinary legs may be replaced by wheels, so that the patient can be carried off without further shifting. The operator is able to alter the height to suit his convenience, and the surface of the table is adjustable at any angle upon turning a winch. Another movement brings up a lattice pillow at a comfortable curve, a great improvement on the old-fashioned straight shoulder board, and one that must have taken no little planning and mechanical skill to have constructed.

There are many other points of interest about the history and arrangements of this hospital, which will make the subject of a future paper.

